# Paranasal Sinus Invasion Should Be Classified as T4 Disease in Advanced Nasopharyngeal Carcinoma Patients Receiving Radiotherapy

**DOI:** 10.3389/fonc.2020.01465

**Published:** 2020-11-06

**Authors:** Yajie Zhao, Qin Zhou, Na Li, Liangfang Shen, Zhanzhan Li

**Affiliations:** ^1^ Department of Nuclear Medicine, Xiangya Hospital, Central South University, Changsha, China; ^2^ Department of Oncology, Xiangya Hospital, Central South University, Changsha, China

**Keywords:** nasopharyngeal carcinoma, paranasal sinus, prognosis, staging system, Cox regression

## Abstract

In this study, we explored the association between paranasal sinus invasion and prognosis in patients with advanced nasopharyngeal carcinoma (NPC, (T3/T4N0–3M0), and we assessed the possibility of considering paranasal sinus invasion a T category in the 8th edition of the American Joint Committee on Cancer staging system. We enrolled 352 NPC patients who received intensity-modulated radiotherapy between 2008 and 2012. Clinical characteristics and follow-up data were collected. The incidence of paranasal sinus invasion was 36.4% (128 of 352 patients). Multivariate cox regression analysis indicated that paranasal sinus invasion and cervical lymphatic metastasis were independent negative prognostic factors for overall survival (OS, *P*=0.024, *P*=0.012), progression-free survival (PFS, *P*=0.007, *P*=0.007), and distant metastasis-free survival (DMFS, *P*=0.001, *P*=0.000). The gross tumor volume of the nasopharynx was an independent negative prognostic factor for OS (*P*=0.013). Cox regression analysis indicated that there were no significant differences in OS, PFS, DMFS, or local relapse-free survival (LRFS) between NPC patients with T4 stage disease and those with T3 and paranasal sinus invasion (*P*>0.05). The updated T + N staging system slightly improved the prediction of LRFS (0.649, 95% CI: 0.553–0.745) in NPC patients compared to the AJCC system (0.640, 95% CI: 0.545–0.736; *P*=0.023). Paranasal sinus invasion is independently associated with a poor prognosis in NPC patients. Thus, we recommend that the AJCC staging system upgrade paranasal sinus invasion to the T4 classification.

## Introduction

Nasopharyngeal carcinoma (NPC) is an epithelial carcinoma. More than 70% of new cases are in east and southeast Asia, among which nearly 48% are in China (1, 2). The molecular mechanisms of NPC remain unclear (3–5). In both the 7th and 8th editions of the American Joint Committee on Cancer (AJCC) tumor-node-metastasis staging system for NPC, paranasal sinus invasion is classified as T3 disease (6, 7). However, the Chinese 2008 staging system classifies paranasal sinus invasion as T4 disease, and this system has been widely used in clinical practice for more than 10 years in southeast China (8, 9). Whether paranasal sinus invasion should be classified as T3 or T4 disease remains a subject of debate. The paranasal sinuses are in the anterior and superior portions of the nasopharynx, which are considered important anatomic landmarks for local tumor extension. Adjacent organs such as the sphenoid and ethmoidal sinuses, which are close to the brainstem and optic nerve, determine and limit the dose of radiotherapy, directly influencing the therapeutic effects in NPC patients. The incidence of paranasal sinus invasion is between 14.9 and 27% (10–12). Tian et al. (10) reported that sphenoid sinus invasion accounts for most cases of paranasal sinus invasion, and paranasal sinus invasion is independently associated with a poor prognosis, including overall survival (OS), distant metastasis-free survival (DMFS), and local relapse-free survival (LRFS). However, the rates of OS and LRFS were better for patients with stage T3 disease. Therefore, the authors recommended considering paranasal sinus invasion as stage T3 disease in the AJCC staging system for NPC. However, several recent studies have reported opposite findings. Wu et al. (12) found that paranasal sinus invasion was not only an independent prognostic factor affecting survival rates but that there was no difference between T3 disease with paranasal sinus invasion and T4 NPC. Zhang et al. (13) showed that invasion of the sphenoid sinus can be classified as T3 disease and ethmoid sinus/maxillary sinus invasion may be classified as T4 stage in the latest (8th edition) AJCC staging system.

Here, we performed a follow-up study of 352 NPC patients with locally advanced disease (T3/4N0–3M0) to evaluate the association between paranasal sinus invasion and prognosis, and we assessed the possibility of considering paranasal sinus invasion a T category in the 8th edition of AJCC, which would be a more accurate classification of this disease.

## Materials and Methods

### Patient Selection

A total of 352 NPC patients were selected according to the following criteria: had non-metastatic T3/T4N0-3 (according to the 8th edition of the AJCC staging system) with histological confirmation, and received definitive radiotherapy with or without other treatments at Xiangya Hospital, Central South University (Changsha, China) between August 2008 and January 2012. This study was approved by the Ethics Committee of Xiangya Hospital of Central South University (No. 2011111087).

### Magnetic Resonance Imaging (MRI)

Two experienced clinicians independently reviewed the MRI results. MRI was performed using a 1.5-T Vision Plus Scanner (Siemens, Erlangen, Germany). After radiotherapy for 3 months, there were still obvious abnormal signal foci in the surrounding soft tissue area, and the residual diameters of the cervical lymph nodes and retropharyngeal lymph nodes were greater than 1 and 0.5 cm, respectively, which could be considered MRI image residual.

### Treatment

#### Radiotherapy

The target volumes included the gross tumor volume of the nasopharynx (GTVnx), gross tumor volume of the lymph node (GTVnd), clinical target volume (CTV) 1, and CTV2. The prescription doses delivered to the GTVnx (PGTVnx), PGTVnd, planned target volume (PTV) 1, and PTV2 were 66–73.92 Gy (33 fractions), 59.6–72.6 Gy (33 fractions), 50.4–66 Gy (33 fractions), and 50.4–61.05 Gy (33 fractions), respectively. The dose limits for organs at risk and the criteria for plan evaluation were based on the recommendations of the Radiation Therapy Oncology Group 0225 (14).

#### Chemotherapy

Neoadjuvant chemotherapy was administered during the wait time for radiotherapy to decrease the size of the large tumors. Concurrent chemotherapy was administered in 331 patients. Adjuvant chemotherapy was administered in patients with N2/N3 disease and those with detectable residual tumor at the end of radiotherapy. All chemotherapy regimens were platinum-based. The chemotherapy regimens included 120 mg/m^2^ taxol on day 1 + 80 mg/m^2^ cisplatin on day 2 for 3 weeks/cycle, 1 g/m^2^ gemcitaine on day 1 + 80 mg/m^2^ cisplatin on day 1 for 3 weeks/cycle, and a 4 g/m^2^ 5-FU continuous infusion > 96 h + 80 mg/m^2^ cisplatin on day 1 for 3 weeks/cycle; all treatments were 2–6 cycles.

#### Target Treatment

Target treatment was administered in patients with AJCC stage IVa who could afford the treatment. Twenty patients received targeted therapy, which included nimotuzumab and cetuximab. Nimotuzumab was administered at a dose of 100–200 mg/week. Meanwhile, cetuximab was also administered (initial dose of 400 mg/m^2^, followed by 250 mg/m^2^/week). Sensitization treatment was mainly radiotherapy sensitizer (sodium glycididazole). The usage frequency was according to the guidelines.

### Follow-Up

Regular follow-up was performed *via* telephone or by extracting information from medical records. The time intervals were every 3 months for the first 2 years, and every 6 months thereafter. All patients were seen by ear, nose, and throat (ENT) specialists at the Department of ENT as well as oncologists for consultation and physical examinations, which included MRI of the nasopharynx and neck region, chest X-ray, abdominal ultrasound, and a whole-body bone scan. Details of the follow-up duration and calculation methods, such as distance from the brain stem (according to the anatomic location on computed tomography-MRI fusion images), distance from the edge of the primary tumor to the edge of the brain stem, and residual volume (measured according to the abnormal signal intensity area on MRI after radiotherapy), were previously defined (9, 15). Four different outcomes were recorded.

### Statistical Analysis

All statistical analyses were performed using the Statistical Package for Social Sciences version 23.0 and R software. Quantitative data are expressed as the mean ± standard deviation, and comparisons between groups were performed using the *t*-test. Qualitative data are expressed as the count and percentage. Comparisons of clinical characteristics between the paranasal sinus invasion and non-paranasal sinus invasion groups were performed with the χ^2^ test. Kaplan–Meier curves and log-rank tests were used for comparisons of OS, PFS, LRFS, and DMFS within different categories. Univariate and multivariate analyses of the clinical parameters for OS, PFS, LRFS, and DMFS were conducted using the Cox proportional hazards model. The c-index was also calculated using R 3.6.0 software. The *P*-value of the c-index was obtained using the two-tailed paired *t*-test, calculated according to the rcorrp.cens function in the Hmisc package (16, 17). *P*<0.05 was considered statistically significant.

## Results

### Characteristics of Patients With Paranasal Sinus Invasion

**Figure 1:**
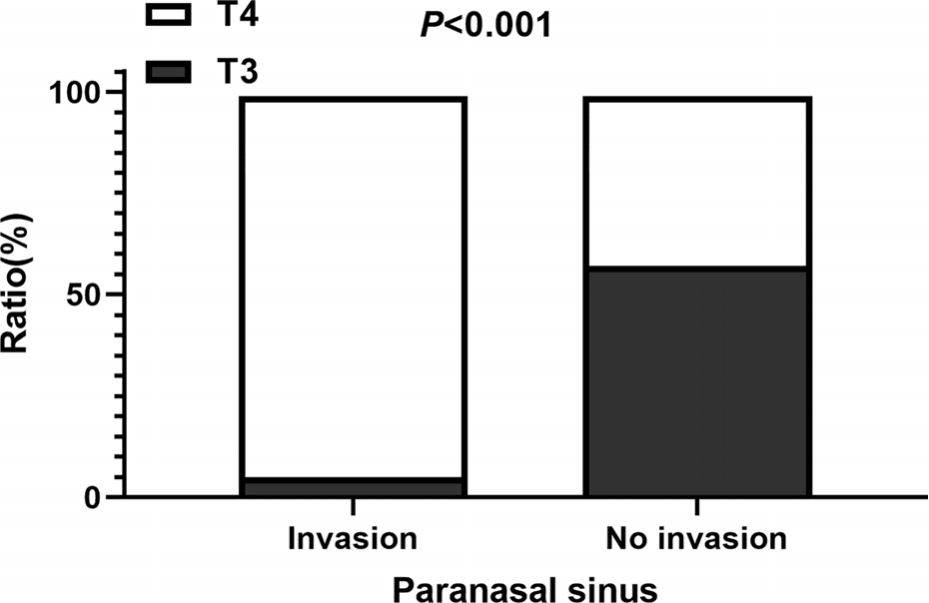
The ratio of patients with T3 and T4 among those with and without paranasal sinus invasion.

The study included 352 NPC patients (251 males and 101 females). The median follow-up time was 34 months (range, 3–66 months). The mean age was 47.2±11.9 years old. The mean Karnofsky Performance Score was 85.9±5.1. There are 329 patients with low differentiation type (93.5%) and 23 with high differentiation type (6.5%); 135 patients (38.4%) had T3 disease and 217 had T4. The number of patients with N classification (N0–N3) was 43 (12.2%), 88 (25.0%), 138 (39.2%), and 83 (23.6%), respectively. In all, 94.0% of patients received chemotherapy, 43.5% received radiosensitizer, and 8.5% received targeted therapy; 15.3% of patients had a cervical lymphatic metastasis and 34.1% had residual nasopharyngeal carcinoma. The incidence of paranasal sinus invasion was 36.4% (128 of 352 patients). The status of paranasal sinus invasion and clinical characteristics of the patients are presented in **Table 1**. The number of patients with paranasal sinus invasion in the T3 and T4 disease groups was 7 (5.1%) and 121 (55.8%), respectively (**Figure 1**). Compared with the non-paranasal sinus invasion group, there was a significant difference between the two groups (*P*<0.001). Patients with sinus invasion tended to be in the advanced T stage (*P*=0.000), had the primary tumor located close to the brain stem (*P*=0.000), and had a higher rate of residual nasopharyngeal carcinoma (*P*=0.000) and GTVnd residual volume (*P*=0.000). There were no significant differences in patient age (*P*=0.726), sex (*P*=0.859), chemotherapy (*P*=0.444), induction therapy (*P*=0.690), concurrent therapy (*P*=0.180), adjuvant chemotherapy (*P*=0.659), radiosensitizer (*P*=0.416), capsular invasion (*P*=0.360), and GTVnx residual volume (*P*=0.129) between the two groups.

**Table 1:** Association between paranasal sinus invasion and clinical parameters in nasopharyngeal carcinoma patients.

Parameters	Paranasal sinus invasion		t/χ^2^	*P*
	Yes	No		
Age (year)	47.5±11.7	47.0±12.0	0.350	0.726
Gender (male, %)	159(71.0%)	92(71.9%)	0.032	0.859
KPS score	85.7±4.9	85.9±5.1	-0.419	0.676
Histological type (WHO) (n, %)			1.397	0.237
I	11(8.6%)	12(5.4%)		
II & III	117(91.4%)	212(94.6%)		
T classification			91.993	0.000
T3	7(5.5%)	128(57.1%)		
T4	121(94.5%)	96(42.9%)		
N classification			8.304	0.040
N0	21(16.4%)	22(9.8%)		
N1	39(30.5%)	49(21.9%)		
N2	42(32.8%)	96(42.9%)		
N3	26(20.3%)	57(25.4%)		
Chemotherapy (n, %)	122(95.3%)	209(93.3%)	0.586	0.444
Induction	116(90.6%)	200(89.3%)	0.159	0.690
Concurrent	78(60.9%)	120(53.6%)	1.796	0.180
Adjuvant	92(71.9%)	156(69.6%)	0.195	0.659
Radiosensitizer (n, %)	52(40.6%)	101(45.1%)	0.661	0.416
Targeted therapy (n, %)	16(12.5%)	14(6.3%)	4.081	0.043
Cervical lymphatic metastasis (n, %)	12(9.4%)	42(18.8%)	5.512	0.019
Capsular invasion (n, %)	6(4.7%)	16(7.1%)	0.838	0.360
Distance from the brain stem	4.3±3.3	7.5±3.1	9.130	0.000
Nasopharynx residue	59(46.1%)	61(27.2%)	12.897	0.000
GTVnx residue volume (ml)	0.3±1.2	0.7±2.4	1.523	0.129
GTVnd residue volume(ml)	6.9±11.7	2.6±6.1	4.043	0.000

KPS, Karnofsky percent score

Histological type (WHO) of Ⅰ, Ⅱ, Ⅲ are keratinizing squamous, non-keratinizing, and basaloid squamous respectively.

### Prognosis of Patients With Paranasal Sinus Invasion

**Figure 2:**
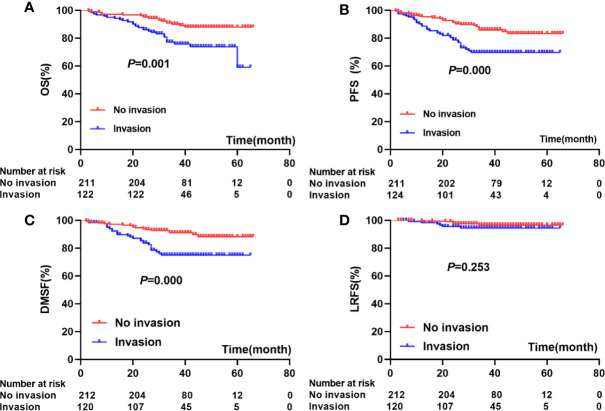
Association between paranasal sinus invasion and prognosis: **(A)** (OS), **(B)** (PFS), **(C)** (DMFS), **(D)** (LRFS).

Compared to patients without paranasal sinus invasion, patients with paranasal sinus invasion had longer 5-year OS (87.9% vs. 59.1%; *P*=0.001), PFS (89.7% vs. 70.9%; *P*=0.000), and LRFS (96.7% vs. 94.4%; *P*=0.000); no significant difference was found in DMFS (88.3% vs. 75.0%; *P*=0.253) (**Figure 2**). As shown in **Table 2**, univariate Cox regression analysis indicated that paranasal sinus invasion was associated with a worse prognosis in NPC patients: OS (hazard ratio [HR]: 2.60, 95% CI: 1.46–4.62; *P*=0.001), PFS (HR: 2.36, 95% CI: 1.44–3.86; *P*=0.001), and DMFS (HR: 2.76, 95% CI: 1.53–4.96; *P*=0.001). Patients with T4 disease had poorer OS (HR: 3.24, 95% CI: 1.52–6.93; *P*=0.002), PFS (HR: 2.39, 95% CI: 1.32–4.33; *P*=0.004), and DMFS (HR: 3.19, 95% CI: 1.49–6.82; *P*=0.003) compared to patients with T3 disease but not RFS. Cervical lymphatic metastasis was also negatively associated with OS (HR: 2.19, 95% CI: 1.16–4.16; *P*=0.016), PFS (HR: 2.31, 95% CI: 1.32–4.02; *P*=0.003), and DMFS (HR: 3.76, 95% CI: 2.06–6.86; *P*=0.000). Patients with GTVnx-residual had poorer OS (HR: 2.53, 95% CI: 1.35–4.47; *P*=0.004) and PFS (HR: 1.81, 95% CI: 1.08–3.02; *P*=0.023), and GTVnd-residual was only associated with OS (HR: 2.57, 95% CI: 1.45–4.54; *P*=0.001) and DMFS (HR: 1.84, 95% CI: 1.02–3.32; *P*=0.044). **Table 3** presents the results of multivariate Cox regression analysis for the prognosis of NPC patients, which indicated that paranasal sinus invasion was associated with a poor prognosis in NPC patients: OS (HR: 2.11, 95% CI: 1.10–4.03; *P*=0.024), PFS (HR: 2.10, 95% CI: 1.18–3.76; *P*=0.012), and DMFS (HR: 2.59, 95% CI: 1.30–5.16; *P*=0.007). Cervical lymphatic metastasis was also negatively associated with OS (HR: 2.49, 95% CI: 1.29–4.81; *P*=0.007), PFS (HR: 2.74, 95% CI: 1.54–4.88; *P*=0.001), and DMFS (HR: 5.01, 95% CI: 2.67–9.40; *P*=0.000). Patients with GTVnx-residual had a poorer OS (HR: 2.22, 95% CI: 1.18–4.18; *P*=0.013). Multivariate analysis showed that cervical lymphatic metastasis and paranasal sinus invasion were independent negative prognostic factors for OS, PFS, and DMFS (*P*=0.007, *P*=0.001, *P*=0.000, and *P*=0.024, *P*=0.012, *P*=0.007, respectively). GTVnx-residual was an independent negative prognostic factor for OS (*P*=0.013; **Table 3**).

**Table 2:** Univariate cox regression of relationship between clinical parameters and survival status in patients with NPC.

Parameters		OS		PFS		DMFS		LRFS	
		HR (95%CI)	*P*	HR (95%CI)	*P*	HR (95%CI)	*P*	HR (95%CI)	*P*
Age	<60	1.00		1.00		1.00		1.00	
	≥60	1.66(0.83-3.33)	0.155	1.11(0.57-2.18)	0.759	1.11(0.50-2.49)	0.792	2.03(0.55-7.50)	0.288
Gender	Female	1.00		1.00		1.00		1.00	
	Male	1.51(0.83-2.73)	0.175	1.04(0.60-1.80)	0.880	1.18(0.63-2.20)	0.614	0.91(0.25-3.37)	0.891
KPS score	<85	1.00		1.00		1.00		1.00	
	≥85	0.95(0.53-1.67)	0.848	0.78(0.48-1.28)	0.330	0.90(0.50-1.62)	0.731	0.50(0.16-1.58)	0.238
Histological type (WHO)	I	1.00		1.00		1.00		1.00	
	II & III	1.86(0.74-4.69)	0.190	1.58(0.68-3.67)	0.284	1.45(0.52-4.06)	0.475	3.15(0.69-14.36)	0.139
T classification	T3	1.00		1.00		1.00		1.00	
	T4	**3.24(1.52-6.93)**	**0.002**	**2.39(1.32-4.33)**	**0.004**	**3.19(1.49-6.82)**	**0.003**	3.31(0.73-15.13)	0.122
N classification	N0	1.00		1.00		1.00		1.00	
	N1	2.03(0.67-6.12)	0.211	2.02(0.67-6.12)	0.211	0.98(0.33-2.92)	0.968	0.78(0.13-4.69)	0.789
	N2	0.94(0.29-2.99)	0.911	0.94(0.29-2.99)	0.911	0.82(0.29-2.35)	0.717	0.97(1.19-4.84)	0.974
	N3	2.83(0.96-8.34)	0.601	2.83(0.96-8.34)	0.060	2.41(0.90-6.45)	0.080	0.26(0.02-2.88)	0.273
Chemotherapy	Yes	1.00		1.00		1.00		1.00	
	No	0.66(0.24-1.84)	0.425	0.58(0.25-1.34)	0.198	0.84(0.26-2.71)	0.771	0.29(0.06-1.31)	0.107
Radiosensitizer	Yes	1.00		1.00		1.00		1.00	
	No	1.05(0.59-1.88)	0.863	1.08(0.66-1.77)	0.773	1.05(0.58-1.88)	0.880	2.67(0.80-8.88)	0.110
Targeted therapy	Yes	1.00		1.00		1.00		1.00	
	No	1.86(0.83-4.16)	0.131	1.64(0.78-3.44)	0.191	1.71(0.73-4.04)	0.220	0.92(0.12-7.15)	0.939
Cervical lymphatic metastasis	No	1.00		1.00		1.00		1.00	
	Yes	**2.19(1.16-4.16)**	**0.016**	**2.31(1.32-4.02)**	**0.003**	**3.76(2.06-6.86)**	**0.000**	0.49(0.06-3.82)	0.498
Paranasal sinus invasion	No	1.00		1.00		1.00		1.00	
	Yes	**2.60(1.46-4.62)**	**0.001**	**2.36(1.44-3.86)**	**0.001**	**2.76(1.53-4.96)**	**0.001**	1.91(0.62-5.94)	0.261
Capsular invasion	No	1.00		1.00		1.00		1.00	
	Yes	1.42(0.51-3.97)	0.500	0.76(0.24-2.42)	0.643	0.35(0.05-2.50)	0.293	0.05(0-21.01)	0.574
Distance from the brain stem	<6.5	1.00		1.00		1.00		1.00	
	≥6.5	0.58(0.32-1.05)	0.072	0.66(0.40-1.09)	0.105	0.75(0.42-1.34)	0.330	1.07(0.35-3.32)	0.904
Nasopharynx residue	No	1.00		1.00		1.00		1.00	
	Yes	1.14(0.63-2.06)	0.673	1.32(0.80-2.19)	0.276	1.08(0.59-1.99)	0.804	1.95(0.63-6.04)	0.250
GTVnx residue	No	1.00		1.00		1.00		1.00	
	Yes	**2.53(1.35-4.47)**	**0.004**	**1.81(1.08-3.02)**	**0.023**	1.54(0.85-2.79)	0.156	1.40(0.42-4.64)	0.586
GTVnd residue	No	1.00		1.00		1.00		1.00	
	Yes	**2.57(1.45-4.54)**	**0.001**	1.52(0.90-2.54)	0.114	**1.84(1.02-3.32)**	**0.044**	4.79(1.05-21.93)	0.043

OS, overall survival; PFS, progression-free survival; DMFS, distant metastasis free survival; LRFS, local recurrence-free survival; KPS, Karnofsky percent score.

Bold values mean statistically significant.

**Table 3:** Multivariate cox regression of relationship between clinical parameters and survival status in patients with NPC*.

Parameters		OS		PFS		DMFS		LRFS	
		HR (95%CI)	*P*	HR (95%CI)	*P*	HR (95%CI)	*P*	HR (95%CI)	*P*
T classification	T3	1.00		1.00		1.00		1.00	
	T4	2.16(0.92-5.07)	0.076	1.65(0.83-3.31)	0.156	2.32(0.96-5.61)	0.062	2.15(0.41-11.4)	0.368
Cervical lymphatic metastasis	No	1.00		1.00		1.00		1.00	
	Yes	**2.49(1.29-4.81)**	**0.007**	**2.74(1.54-4.88)**	**0.001**	**5.01(2.67-9.40)**	**0.000**	0.47(0.06-3.67)	0.468
Paranasal sinus invasion	No	1.00		1.00		1.00		1.00	
	Yes	**2.11(1.10-4.03)**	**0.024**	**2.10(1.18-3.76)**	**0.012**	**2.59(1.30-5.16)**	**0.007**	1.08(0.32-3.18)	0.898
GTVnx residue	No	1.00		1.00		1.00		1.00	
	Yes	**2.22(1.18-4.18)**	**0.013**	1.21(0.68-2.14)	0.522	0.78(0.39-1.54)	0.468	4.31(0.86-21.57)	0.075
GTVnd residue	No	1.00		1.00		1.00		1.00	
	Yes	1.32(0.65-2.68)	0.439	1.33(0.76-2.34)	0.321	1.82(0.94-3.54)	0.076	0.90(0.26-3.13)	0.866

*variables that are significant in the univariate cox regression of any outcomes (OS, PFS, DMFS, LRFS) are included in the multivariate regression.

OS, overall survival; PFS, progression-free survival; DMFS, distant metastasis free survival; LRFS, local recurrence-free survival

Bold values mean statistically significant.

### T Classification for Paranasal Sinus Invasion

**Figure 3:**
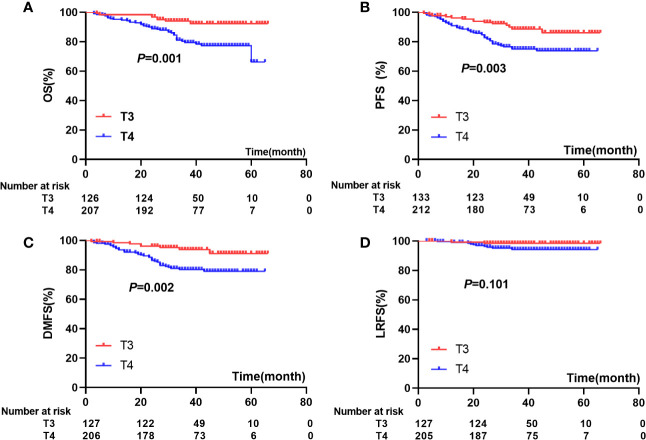
comparisons of prognosis among patients with T3 and T4: **(A)** OS), **(B)** (PFS), **(C)** (DMFS), **(D)** (LRFS).

According to the 8th edition of the AJCC staging system, 135 patients were classified as T3 and 7 (5.1%) developed paranasal sinus invasion; 217 patients were classified as T4 and 121 (55.8%) developed paranasal sinus invasion. The estimated 5-year OS, PFS, LRFS, and DMFS rates for patients with T3 and T4 classification were 92.3% versus 66.2% (*P*=0.001), 86.1% versus 73.9% (*P*=0.003), 91.1% versus 78.9% (*P=*0.002), and 98.4% versus 94.2% (*P*=0.101), respectively (**Figure 3**). Cox regression analysis indicated there were no significant differences in OS, PFS, DMFS, or LRFS between NPC patients with T4 stage disease and those with T3 and paranasal sinus invasion (*P*>0.05; **Table 4**).

**Table 4:** Univariate cox regression of T4 NPC patients compared with those of T3 patients with paranasal sinus invasion.

Survival status	B	SE	Waldχ^2^	P	HR (95%CI)
OS	-0.582	0.725	0.642	0.423	0.56(0.14-2.32)
PFS	-0.465	0.722	0.415	0.519	0.63(0.15-2.58)
DMFS	-0.043	1.013	0.002	0.966	0.96(0.13-6.98)
LRFS	3.043	8.573	0.126	0.723	20.98(0.00-∞)

OS, overall survival; PFS, progression-free survival; DMFS, distant metastasis free survival; LRFS, local recurrence-free survival.

### Upgraded T Staging System

We re-classified the NPC patients with a locally advanced stage. We classified NPC patients with paranasal sinus invasion and T3 stage disease in the T4 group according to the AJCC staging system. We compared the c-indexes of updated and previous classifications using the Cox regression model (**Table 5**). The c-index of the updated T + N staging system slightly improved the prediction of LRFS (0.649, 95% CI: 0.553–0.745) in NPC patients compared to the AJCC system (0.640, 95% CI: 0.545–0.736; *P*=0.023).

**Table 5:** C-index for the AJCC and upgraded staging system in advance NPC patients.

C-index	AJCC	Upgraded	Change(95%CI)	*P*
OS	0.606(0.546-0.666)	0.621(0.566-0.675)	-0.014(-0.045-0.015)	0.343
PFS	0.608(0.556-0.661)	0.619(0.571-0.667)	-0.011(-0.036-0.013)	0.380
DMFS	0.632(0.579-0.685)	0.627(0.570-0.684)	-0.005(-0.031-0.019)	0.680
LRFS	0.640(0.545-0.736)	0.649(0.553-0.745)	0.008(0.001-0.016)	0.023

OS, overall survival; PFS, progression-free survival; DMFS, distant metastasis free survival; LRFS, local recurrence-free survival.

## Discussion

In this study, we found that paranasal sinus invasion and cervical lymphatic metastasis were independent negative prognostic factors for OS, PFS, and DMFS and that GTVnx-residual was an independent negative prognostic factor for OS. Our previous studies confirmed that the distance between the primary tumor and brain stem and residual nasopharyngeal carcinoma after chemotherapy are important factors that affect the survival of NPC patients (17, 18). Here, we found that the OS, PFS, DMFS, and LRFS did not differ between patients with paranasal sinus invasion and T3 disease and those with T4. We upgraded paranasal sinus invasion from T3 to T4 disease, and we found that paranasal sinus invasion can effectively predict the possibility of developing LRFS.

Paranasal sinus invasion in NPC, a local aggressive head and neck malignant tumor, is quite common, especially in patients with locally advanced disease. Both the 7th and 8th editions of the AJCC/UICC staging system have classified paranasal sinus invasion as T3 disease, unlike the Chinese 2008 staging system for NPC (6–8). The correct classification of paranasal sinus invasion is particularly important for NPC patients.

In our study, the incidence of paranasal sinus invasion in NPC patients with T3/4N0–3M0 disease was 36.4%, which is slightly higher than that in other studies showing an incidence between 14.9% and 27% (10–12). This inconsistency is mainly due to the different clinical stages of NPC patients studied. It is well known that paranasal sinus invasion is more common in patients with locally advanced disease than early-stage disease. One reason is that there is no muscle or fascia to act as a barrier to protect the paranasal sinuses directly. In addition, the primary nasopharynx tumor mass in locally advanced disease is larger than that in early-stage disease. Due to the high incidence of NPC, an increasing amount of attention has been paid to the relationship between paranasal sinus invasion and prognosis. It has been shown that paranasal sinus invasion is an independent prognostic factor for OS, DMFS, LRFS, and PFS (10, 12), but the classification of paranasal sinus invasion in current staging systems remains a subject of debate. Tian et al. (10) found that OS and LRFS were significantly better in T3 disease with paranasal sinus invasion than in T4 disease, and they proposed that classification of paranasal sinus invasion as stage T3 was reasonable. However, only 14.9% of patients received intensity-modulated radiation therapy (IMRT) in their study, and most of the patients (80.2%) received two-dimensional radiation therapy. Recently, an increasing number of researchers have analyzed the relationship between paranasal sinus invasion and clinical staging after IMRT in NPC patients. Wu et al. (12) found that paranasal sinus invasion was an independent negative prognostic factor for OS, PFS, LRFS, and DMFS, and the survival of T3 NPC patients with paranasal sinus invasion was similar to that of T4 patients. Thus, they suggested upgrading the T classification of NPC with paranasal sinus invasion to T4. However, the clinical staging used in the current study was the 7th edition of the AJCC staging system for NPC. Zhou et al. (19) confirmed that the 7th edition had some limitations, and adjusted the T category as an independent prognostic factor for OS/DMFS/DFS (with the exception of LRFS). In the present study, we used the 8th edition of the AJCC staging system to confirm the relationship between prognosis and paranasal sinus invasion. Our survival analysis showed no significant difference in OS, PFS, DMFS, and LRFS between T3 NPC patients with paranasal sinus invasion and T4 NPC patients, similar to the study by Cao et al. (19). In their study, the authors recommended that paranasal sinus invasion be classified as T4 in the 8th edition of the AJCC staging system for NPC. However, it has also been suggested by others that it is more reasonable to classify the severity of T staging according to the different sites of paranasal sinus invasion. Zhang et al. (13) suggested that involvement of different paranasal sinuses should be grouped into different T classifications, and that patients with sphenoid sinus invasion alone should be classified as T3 disease.

We found that paranasal sinus invasion was an independent negative prognostic factor for OS, PFS, and DMFS. We also upgraded the T staging system to confirm that paranasal sinus invasion has prognostic value in NPC patients. There were no significant differences in OS, PFS, and DMFS between T3 NPC patients with paranasal sinus invasion and those with T4 disease after upgrading of the T staging system. However, LRFS, an important representation of the T classification, showed a significant difference after the upgrade. Our results are in accordance with those of Wu et al. (12). The authors also reported that the updated T + N staging system slightly improved the prediction ability of OS in NPC patients. Their research further found that T3 patients with paranasal sinus invasion had a poorer prognosis than those without, but paranasal sinus invasion did not affect the prognosis of T4 patients. These results confirm that paranasal sinus invasion affects prognosis in patients with advanced NPC, particularly the LRFS. Therefore, paranasal sinus invasion should be classified as T4 disease in advanced NPC patients.

Our study has several limitations. First, the ratio of censored data was high because NPC patients usually have a long median survival time and longer than 5-year follow-up. However, our data were in accordance with several previous studies, which have also shown a similar and close ratio of censored data. This suggests that some bias existed in our analysis, but the bias was controlled. Second, the sample size of the T3 patients with paranasal sinus invasion was very small, and paranasal sinus invasion was an independent negative prognostic factor for advanced NPC. However, the upgraded AJCC staging system should be further tested in a study with a larger sample size and longer follow-up time. Similarly, we could not perform subgroup analyses for the paranasal sinuses because of the limited sample size.

In conclusion, paranasal sinus invasion appears to be an important negative prognostic factor for advanced NPC. Thus, it may be worth updating the classification of paranasal sinus invasion to T4 in the 8th edition of the AJCC/UICC staging system for NPC. Studies with a larger sample size are required to confirm our findings.

## Data Availability Statement

The raw data supporting the conclusions of this article will be made available by the authors, without undue reservation.

## Ethics Statement

The studies involving human participants were reviewed and approved by Ethics Committee of Xiangya Hospital of Central South University (Number: 2011111087). The ethics committee waived the requirement of written informed consent for participation.

## Author Contributions

ZL and LS designed this experiment and directed the research group in all aspects, including planning, execution, and analysis of the study. YZ as the first author was the main investigator in this study, screening patients, performing follow-up evaluation, statistical analysis, and writing this article. NL and QZ participated in follow-up evaluation and statistical analysis. All authors contributed to the article and approved the submitted version.

## Funding

This work was partly supported by the Hunan Department of Science and Technology Foundation, China (YZ: NO. 2018JJ3827). The achievements of this paper are supported by China Scholarship Council (YZ: NO. 201906375015), the Science Foundation of Xiangya Hospital for Young Scholar (ZL: NO. 2018Q012), National Natural Science Foundation of China (ZL: No. 82003239) and (YZ: 82002887), and Hunan Province Natural Science Foundation (Youth Foundation Project) (ZL: NO. 2019JJ50945).

## Conflict of Interest 

The authors declare that the research was conducted in the absence of any commercial or financial relationships that could be construed as a potential conflict of interest.
